# Prognostic accuracy of clinicians for back, neck and shoulder patients in routine practice

**DOI:** 10.1186/2045-709X-21-42

**Published:** 2013-12-01

**Authors:** Dave Newell, Jonathan Field, Nita Visnes

**Affiliations:** 1 AECC-Anglo European College of Chiropracti, 14-15 Parkwood Road, BH5 2DF, UK; 2 Private practice, Bournemouth, UK; 3 AECC, Bournemouth, UK

**Keywords:** Prognosis, Practitioner, Prediction, Outcome, Chiropractic

## Abstract

**Background:**

Chronicity amongst musculoskeletal patients remains a considerable burden and predicting outcomes in these patients has proven difficult. Although a large number of studies have investigated a range of predictors of outcome few have looked at the practitioners’ ability to discern those that improve from those most likely to fail to improve. This study aimed to investigate the ability of chiropractors to predict patient outcomes.

**Methods:**

Prediction and outcome data were collected from 440 consecutive patients with back, neck or shoulder pain accepted for chiropractic care within 5 linked private practices.

Predictions by chiropractors were compared to patient outcomes as measured by Bournemouth Questionnaire (BQ) scores, pain NRS scores and patient global impression of change (PGIC) collected at 4 and 12 weeks following the initial consultation.

**Results:**

Overall, chiropractors appear unable to accurately predict poor outcomes in their patients particularly in the longer term. Although some conditions (neck) faired a little better in some cases with some trends in short term pain scores being associated with the clinicians prediction, this was marginal. Subgrouping by practitioners or duration did not improve the performance of these predictions

**Conclusions:**

Chiropractors generally fail to reliably predict poor treatment outcome of patients at initial consultation.

## Background

Musculoskeletal disorders (MSD) remain a burden both in terms of suffering and economics with European economies paying up to €240 billion a year in health and work related costs [1]. Up to 80% of the population will suffer from MSDs at some point in their life [2] and at least 100 million people reported chronic musculoskeletal pain in 2008 [3]. Some types of MSD are more prevalent than others. For example, the department of health found that 13-15% of unemployed individuals in Britain between 16 and 64 years of age cite back pain as their reason to be out of work with 5% of employed back pain sufferers having taken time of work in the past month due to pain. It is estimated that annually British businesses lose 4.9 billion work days due to back pain with UK total costs associated with MSDs of around £7 billion [4]. The need to return patients to work and satisfactory resolution of these types of disorders is clearly imperative and The Work Foundation [1] states that *‘return to work and maintaining work productivity should be explicit clinical targets, and in this respect, the benefits of early diagnosis, appropriate intervention and effective rehabilitation in managing MSDs are clear’*.

The ability to identify patients likely to respond differentially to care is important to enable the provision of targeted advice and care [5]. This has been described by the Cochrane collaboration as the number one priority in back pain research [6]. Despite a large number of studies looking at prognostic factors associated with poor outcome in those attending chiropractors, few robust predictors have emerged with controversy surrounding those that have been found [7-9]. However, it is possible that clinicians have an additional insight into the likelihood of recovery or otherwise of MSD patients attending seeking their care. If true practitioners do indeed have an insight into their patients prognosis this could potentially enable an exploration of the factors behind this prognostic ability as a direction for future work.

Previous research suggests the ability of clinicians in general to predict accurately the likely response of patients to care is uncertain [10]. Hill et al. [11] reported that clinicians (General practitioners, physiotherapists and pain management specialists) using intuition alone to make risk estimations for LBP patients had little agreement compared to a formal screening tool (Start Back Tool) and low inter-clinician agreement. In contrast, Jellema et al. [5] implies that general practitioners ability to assess risk does not dramatically differ from the Örebro Musculoskeletal Pain Screening Questionnaire, the Low Back Pain (LBP) Perception Scale and a clinical prediction rule created for the study. The only other study reporting associations between the clinicians’ prediction and LPB patients’ outcome was where the primary goal of the study was to generate a clinical prediction rule (CPR). Here, the physiotherapist predictions performed poorly in comparison to the CPR which itself generated AUCs considered to poor, despite the clinicians predictions still adding some utility in the final model [12].

Few if any studies have investigated the ability of chiropractors to predict outcomes in their patients. Given that most LBP patients attending chiropractic care report a good outcome, identifying those who will do poorly as early as possible may allow greater utility for clinicians and researchers in providing more targeted approaches [8]. This study aimed therefore to explore how well chiropractors are able to identify which patients are more likely to fail to recover while undergoing a course of chiropractic care.

## Methods

### Subjects

Patients with low back, neck or shoulder pain, over 16 years of age and seeking care for the first time from UK chiropractors and were eligible. Patient consent to use their anonymised data for research purposes was achieved via a tick box during routine on line collection of clinical baseline and outcome reporting. The chiropractor’s experiences ranged from 2 to 20 years and were aged between 24 and 45 with two being female. Inclusion criteria were; accepted for chiropractic treatment and consented to being sent e-mails as part of an electronic patient reported outcome measures (Care Response) system in these clinics.

### Procedure

Chiropractors were asked to record on a form (immediately after the first consultation) whether they thought patients were less likely than average to report a good outcome following a course of care. Patient data collected as normal practice activity was analysed. The data collected at baseline on paper forms at the practice included gender, age (years) , complaint (body area diagram), and duration of complaint (less than 4 weeks, less than 4 weeks recurring and greater than 4 weeks), total Bournemouth Questionnaire (BQ) [13] scores (0–70;higher is worse), pain NRS (0–10) and practice attended.

Patients were then further contacted by either post or email with requests to complete the BQ, pain NRS and patient global impression of change (PGIC) questionnaires at 4 week and 12 week follow up. The PGIC consisted of a question and 6 possible responses as follows:

“How would you describe your pain/complaint now, compared to how you were when you completed the questionnaire before your first visit to this clinic?”

ϑ Very much improved

ϑ Much improved

ϑ Slightly improved

ϑ No change

ϑ Slightly worsened

ϑ Much worsened

ϑ Worse than ever

The setting was 5 UK clinics and involved the practices of 6 separate chiropractors.

### Data analysis

We used a primary (BQ Total) and two secondary (Pain NRS, PGIC) measures of outcome and chose to treat the chiropractors predictions as a univariate predictive model, calculating, odds ratios for not improving if the chiropractor assigned a likelihood of poor recovery, positive and negative likelihood ratios, the percentage of variation in outcome explained by the model (Nagelkerke) and area under the curve (AUC) via ROC analysis. This involved using chiropractors’ baseline classification of patient status against status of the patient as defined by the outcomes at 4 and 12 week follow-up points. Area under the curve figures were interpreted as follows; .90-1 = excellent, .80-.90 = good, .70-.80 = fair, .60-.70 = poor, .50-.60 = fail.

Categorisation of patient self-reported status at 4 and 12 week follow up was determined by using the reported minimal clinically important change for the total BQ as a cut off for non-improvement for back (≤ 46%) [14] and neck (≤ 35%) pain [15]. For the pain NRS a cut off of ≤29% [16] indicated non improvement while for the PGIC a cut off of <6 points indicated non improvement [7]. Subgroup analysis was carried out where numbers allowed, using; patient complaint, chiropractic practice and complaint duration as stratifying variables. All analysis was carried out using SPSS (v21).

### **Ethics**

This research was ethically approved by the Anglo European College of Chiropractic undergraduate ethics panel in May 2011.

## Results

Four hundred and forty patients with complete data were included in the analysis at baseline. Respondents at 4 and 12 week follow up were 255 (58%) and 182 (41%) respectively. Tables 1 and 2 illustrate the demographic characteristics and baseline/follow up scores respectively. Back and neck pain patients showed similar profiles in terms of duration and baseline pain and BQ scores. In contrast most shoulder pain patients had higher chronicity.

**Table 1:** Demographic characteristics split by complaint

**Variable**	**Back**	**Neck**	**Shoulder**
Age (Mean (SD))	44.0 (15.0)	45.7 (15.5)	45.5 (15.9)
Gender (% Female)	52	51	46
Treatments at 1 month	4.6 (1.7)	4.6 (1.5)	4.6 (1.4)
Duration (%)			
< 1 month	31	32	17
< 1 month recurring	23	21	17
> 1 month	46	46	65

**Table 2:** Baseline and follow up measures split by complaint

	**Back**	**Neck**	**Shoulder**
**Baseline (n=440)**	**n=312**	**n=71**	**n=57**
Pain NRS (Mean (SD))	5.9 (2.2)	5.6 (2.4)	5.7 (2.3)
BQ total (Mean (SD))	36.3 (15.1)	34.2(16.6)	32.7 (14.9)
**Week 4 (n=255)**	**n=173**	**n=45**	**n=37**
Pain NRS (Mean (SD))	2.1 (2.0)	1.8 (2.0)	2.2 (1.8)
BQ total (Mean (SD))	11.9 (13.3)	11.8 (11.4)	11.3 (10.6)
**Week 12 (n=182)**	**n=124**	**n=35**	**n=23**
Pain NRS (Mean (SD))	2.1 (2.4)	1.6 (2.1)	1.2 (1.8)
BQ total (Mean (SD))	12.2 (14.0)	10.4 (12.9)	7.9 (10.4)

Table 3 illustrates the mean percentage change of the continuous outcome measures between baseline and follow up points. Generally, a greater than 50% drop in total BQ and pain scores are observed in all 3 conditions at 4 weeks follow up. This deteriorates slightly for back pain patients, plateaus for neck pain and substantially further improves for shoulder pain at 12 weeks follow up. Changes both in total BQ and pain scores show similar values with changes in pain being generally higher that changes in total BQ with shoulder pain patients improving the most.

**Table 3:** Change (%) in BQ total and pain NRS scores split by complaint

	**Back**	**Neck**	**Shoulder**
**Week 1- 4**	**n=173**	**n=45**	**n=37**
BQ total	62.4 (42.2)	62.7 (32.1)	56.9 (40.5)
Pain NRS	62.8 (36.5)	65.3 (37.0)	58.6 (39.7)
**Week 1- 12**	**n=124**	**n=35**	**n=23**
BQ total	59.6 (53.3)	67.5 (34.8)	74.3(31.3)
Pain NRS	56.3 (62.1)	68.0 (41.7)	81.2 (26.3)

Table 4 illustrates these changes as dichotomised around the measures respective MCIC values. For both dichotomised pain and total BQ, similar proportions of LBP patients failed to improve at both 4 and 12 week follow up with around 20 to 25% not improving at 12 weeks. A lower proportion of around 10% fail to improve in the neck pain group by the same time. Of note is the fact that nearly all of the shoulder patients improved by the 12th week with only 4% not improving, although this was only calculated with dichotomous pain scores as there is no validated MCIC for the BQ and shoulder pain.

**Table 4:** Proportion (%) of patients not improving as defined by cut off values for BQ total, pain NRS and PGIC scores split by complaint

	**Back (n=nI/I)**	**Neck (n=nI/I)**	**Shoulder (n=nI/I)**
BQ total*			
Week 4	26.3 (45/128)	21.2 (9/36)	nc
Week 12	26.8 (33/91)	14.3 (5/30)	nc
Pain NRS**			
Week 4	17.4 (29/143)	15.9 (7/38)	16.7 (6/30)
Week 12	20.2 (25/99)	11.8 (4/31)	4.3 (1/22)
PGIC***			
Week 4	30.6 (53/120)	26.7 (12/33)	37.8 (14/23)
Week 12	27.4 (34/90)	22.9 (8/27)	21.7 (5/18)

*(≤ 47% (Back), ≤ 36% (Neck)), **(≤ 30%), ***(≤ 5 points), nc=not calculated as no MCIC, nI/I= not improved/Improved.

In slight contrast, the proportions of patients failing to improve as defined by the dichotomised PGIC were somewhat higher for all conditions being around a third at 4 weeks. These proportions remained similar at 12 weeks for back pain, falling slightly for neck pain and dramatically for shoulder pain following a similar pattern to the previous dichotomised outcomes.

Tables 5, 6, 7 report the analysis of the accuracy of the clinicians judgment in correctly identifying those patients failing to improve at 4 and 12 weeks follow up, as defined by 3 outcomes, total BQ, NRS pain and PGIC, dichotomised at their respective MCICs.

**Table 5:** Utility of chiropractors’ prediction of patients not improving as categorised by MCIC in BQ total* scores split by complaint

	**Back**	**Neck**	**Shoulder**
**Week 4**			
**N**	**173**	**45**	
OR (95% CI)	1.7 (0.8 to 3.8)	3.2 (0.7 to 15.0)	nc
Nagelkerke R^2^	0.01	0.07	nc
+ve Likelihood ratio	0.0	0.0	nc
−ve Likelihood ratio	**	**	nc
AUC	0.5 (0.4 to 0.6)	0.6 (0.4 to 0.8)	nc
**Week 12**			
**N**	**124**		
OR (95% CI)	1.7 (0.6 to 4.9)	§	nc
Nagelkerke R^2^	0.01	§	nc
+ve Likelihood ratio	0.0	§	nc
−ve Likelihood ratio	**	§	nc
AUC	0.5 (0.4 to 0.7)	§	nc

*(≤ 47% (Back), ≤ 36% (Neck)), nc=not calculated as no MCIC, **unable to calculate as divided by 0, § unable to calculate due to less than 5 in one cell, AUC=Area under curve.

**Table 6:** Utility of chiropractors’ prediction of patients not improving as categorised by MCIC in pain NRS* scores split by complaint

	**Back**	**Neck**	**Shoulder**
**Week 4**			
**N**	**173**	**45**	**37**
OR (95% CI)	1.3 (0.5 to 3.3)	1.4 (0.2 to 8.9)	5.5 (0.8 to 36.0)
Nagelkerke R^2^	0.00	0.01	0.15
+ve Likelihood ratio	0.0	0.0	0.0
−ve Likelihood ratio	**	**	**
AUC	0.5 (0.4 to 0.6)	0.5 (0.3 to 0.8)	0.7 (0.5 to 0.9)
**Week 12**			
**N**	**124**		
OR (95% CI)	3.1 (1.1 to 9.3)	§	§
Nagelkerke R^2^	0.05	§	§
+ve Likelihood ratio	0.0	§	§
−ve Likelihood ratio	**	§	§
AUC	0.6 (0.4 to 0.7)	§	§

*(≤ 30%), nc=not calculated as no MCIC, **unable to calculate as divided by 0, § unable to calculate due to less than 5 in one cell, AUC=Area under curve.

**Table 7:** Utility of chiropractors’ prediction of patients not improving as categorised by PGIC* scores split by complaint

	**Back**	**Neck**	**Shoulder**
**Week 4**			
**N**	**173**	**45**	**37**
OR (95% CI)	1.1 (0.5 to 2.5)	4.0 (0.9 to 17.8)	1.3 (0.3 to 5.2)
Nagelkerke R^2^	0.00	0.10	0.00
+ve Likelihood ratio	0.0	2.8	0.0
−ve Likelihood ratio	**	0.7	**
AUC	0.5 (0.4 to 0.6)	0.6 (0.4 to 0.8)	0.5 (0.3 to 0.7)
**Week 12**			
**N**	**124**	**35**	**23**
OR (95% CI)	2.2 (0.8 to 6.1)	0.5 (0.05 to 4.9)	0.5 (0.04 to 5.5)
Nagelkerke R^2^	0.03	0.02	0.02
+ve Likelihood ratio	0.0	0.0	0.0
−ve Likelihood ratio	**	**	**
AUC	0.6 (0.4 to 0.7)	0.5 (0.3 to 0.8)	0.6 (0.3 to 0.8)

*(≤ 5 points), **unable to calculate as divided by 0, AUC=Area under curve.

In general both the ability of the clinician to predict outcome (Odds Ratio (OR), Nagelkerke) and the discriminative ability of this prediction in separating those patients that did not improve from those that did (+ve and -ve Likelihood ratio, AUC) were extremely poor regardless of the outcome measure used. Although there were some notable minor improvements in these values for the short term (4 weeks) prediction of neck and shoulder patients, the likelihood ratios still indicated no discriminative power, with AUCs still falling into the ‘poor’ or ‘fair’ category. All other AUC values are considered to be in a ‘failed’ category in their ability to predict or discriminate actual outcomes. Interestingly, the mean NRS continuous scores were significantly higher at 4 weeks follow up in the non-improved compared to the improved patients as categorised by the clinician at baseline. However, the PGIC at 4 weeks follow up and both the NRS and PGIC continuous scores at 12 weeks follow up were no different between the predicted categories.

When the primary outcome scores were stratified by the individual practitioners or the duration of the condition this predictive and discriminative ability was no better than the previous analysis with all the AUC values falling into the ‘failed’ category apart from a single practitioner who scored ‘poor’ at 4 and 12 weeks. Because of the reduction in numbers of patients failing to improve due to stratification of the BQ scores to less than 5 individuals in some cases we performed the same analysis using the secondary PGIC outcome which had larger numbers of non-improved patients at each time point. This analysis found the same lack of predictive and discriminative power of the practitioners initial judgement found using the primary measure.

## Discussion

Analysis of the ability of chiropractors to predict their own patents’ outcome status suggests that practitioners are overall at best poor and at worst fail. Given that prognosis in this condition does not involve a life threatening outcome, one might argue that the AUC categorisation of the discriminative performance of clinicians might be more lenient, i.e. ‘poor’ becomes ‘fair’ and ‘fail’ becomes ‘poor’. Despite this, the accuracy of the initial clinician predictions remains predominantly poor. The only other studies to investigate clinician prediction of recovery from low back pain stated that GP’s risk estimation was comparable to other prognostic indicators as measured at baseline, although the AUC was reported as only 0.6 and physiotherapists were poorer than a clinical prediction rule which itself scored as poor in terms of AUCs [6,12]. Most other studies investigating prognostic accuracy of physicians have centred on cancer survival with a large systematic review finding only weak evidence to support clinician’s estimates alone as predictors of survival [17]. Predicting other important health outcomes also appears difficult with a recent study investigating the prognostic accuracy of occupational therapist advice regarding return to work times revealing consistent and marked underestimation of recovery by these health workers [18]. However, the literature is not unanimous in its lack of support of clinician based prediction. For example, Reiso et al. [19] found that GPs ability to predict the period of certified sickness absence was high and good prediction was most strongly associated with type of diagnosis. However, the frequent lack of definitive diagnoses in the conditions dealt with in this study, most being categorised as nonspecific, has made prognosis considerably more problematic. Additionally, that the duration of sick certification investigated by the study was potentially under the control of the GP, could be considered a confounding factor. Although a small number of factors associated with poor prognosis have arisen from the MSD literature, particularly low back pain studies, they fail to explain much of the variance reported in outcome. In particular, even fewer robust indicators of prognosis have arisen amongst patients seeking manual therapy and it may not be so surprising why the clinicians in this study struggle to accurately judge outcomes amongst their patients given that extensive research into potential predictive factors of outcome have found so few in this particular MSD population.

Of those that have been reported, reviews of prospective studies reveal a variety of prognostic factors. For example longer pain duration has emerged as a generic prognostic factor amongst MSD patients generally and in low back pain patients in particular [20-22]. However, in this study practitioners were no more accurate in predicting outcomes in chronic (> 1 month) as compared to acute (< 1 month) patients. In addition many studies have indicated psychological factors as important in prognosis of MSD and this is may also be true of chiropractic patients [23] although this remains a matter of controversy [8]. Other factors such as socioeconomic, gender, age and activity have been less reliably related to prognosis in neck pain, with research being indecisive in particular regarding age as a risk factor for poor prognosis [24].

Of interest beyond the primary question of this study are the differences and similarities between the method of determining outcomes and the outcomes of the conditions studied. Generally both dichotomised BQ and pain NRS based determination of improvement or otherwise produce similar proportions of patients at both follow up points. Of note is the fact that after 12 weeks around one 20 to 25% of patients remained unimproved for back and neck pain patients. This concurs with previous research that notes that, contrary to commonly held notions, a significant proportion of these patients do not recover entirely [25]. On the other hand those presenting with shoulder pain and in this study, more chronic shoulder pain, seemed to recover remarkably well with the proportion categorised as not improved continuing to fall significantly beyond the first month, unlike with back and neck pain.

Interestingly, the PGIC global measure consistently categorised a greater proportion of patients as not improved at both follow up points across all conditions compared to BQ and pain NRS categorisations. It is possible this may reflect the way this measure may be thought about by patients where it allows any number of factors to be brought into a patients’ judgement of their improvement as opposed to a single measure such as pain or even a multidimensional measure such as the BQ. These differences may certainly warrant further investigation.

There are clear limitations to this study. Firstly, the question we asked the practitioners was ‘Whether they thought patients were less likely than average to report a good outcome following a course of care’. In meetings with the practitioners involved prior to the study this judgement was discussed in relation to patients’ response on the BQ, as the practitioners were familiar with the routine use of this questionnaire in their practice on a day to day basis. However, the question did not explicitly highlight a particular outcome measure. In order to increase the robustness of our conclusion, we therefore used 3 outcome measures dichotomised around published cutoff scores, with the BQ as the primary outcome. Given that similar if not identical findings were generated from all 3 outcomes it would tend to support the conclusion that practitioners fail to predict patient outcome and is less likely to be an idiosyncrasy of the outcome measure we used or a mismatch between the practitioners perception of the original question and the final outcome measure.

Secondly, we chose to analyse the association between practitioner prediction and patient self-reported outcome in a manner reminiscent of diagnostic test validity despite the fact that this was a prospective study. Normally, diagnostic test validity studies would ideally require minimal time periods between gold standard and new test data collection. In view of this, we also calculated the risk of improvement based on the chiropractor’s initial prognosis, typically a method appropriate to prospective studies. Although this provides a further measure of association, risk normally implies some causative impact of the risk factor on the outcome, whereas in our case there is no expectation that the practitioner’s prognosis would impact the actual outcome, although we did not know whether the practitioner had explicitly stated their prognosis to the patients and it is possible that if they had done so, this may have influenced outcome.

Thirdly, chiropractors in this study were not asked to predict patients’ reports of their outcome at any specific time point but in general and it is possible that had they been asked specifically how patients may report themselves at 1 or 3 months, prognostic accuracy would have been found to be higher.

Lastly, this study used outcome data collected as a normal part of practice activity returned by post or email by patients. It is not possible to exclude the possibility that those who did not respond to the request to complete the PROMs would have answered differently. However the proportion reporting a good outcome in this sample is similar to other studies from this group of practices which achieved a higher response rate by including a telephone follow up of non-responders making it less likely that the results quoted here are subject to non-response bias [26].

## Conclusion

In this study chiropractors were found not to be able to accurately predict treatment outcomes of patients prior to treatment at 1 or 3 months follow up, for any of the conditions, chronicity of condition and regardless of the use of multiple measures to determine outcome. The results of this study imply that practitioners insight into a patients likely outcome is not sufficient alone as a prognostic tool. Given the controversy inherent in the prediction literature to define robust predictors of prognosis it maybe that barring generic factors such as duration it will remain up to the practitioner to do their best in how they articulate potential prognosis to the patient. Luckily given that most MSD patients tend to improve in the relatively short term erring on the side of optimism may be the best policy.

## Competing interests

The authors declare that they have no competing interests.

## Authors’ contributions

DN and JF conceived of the study. NV and JF collected the data and contributed to the final manuscript. DN analysed the data, wrote the draft and edited the final manuscript. All authors read and approved the final manuscript.
